# Supplementary material for: Limited Lactosylation of Beta-Lactoglobulin from Cow’s Milk Exerts Strong Influence on Antigenicity and Degranulation of Mast Cells
Source: Nutrients. 2021 Jun 15;13(6):2041. doi: 10.3390/nu13062041 (PMC8232271; doi:10.3390/nu13062041)
Supplement: Supplementary file 1 [file nutrients-13-02041-s001.zip › nutrients-1252243-SI.pdf]

Supplementary information;

Limited lactosylation of beta-lactoglobulin from cow's milk exerts strong influence on antigenicity and allergenicity

Gerlof P. Bosman[1]; Sergio Oliveira[2]; Peter J. Simons[3]; Javier Sastre Torano[1]; Govert Somsen[4]; Leon Knippels[2,5]; Rob Haselberg[4]; Roland J. Pieters[1], Johan Garssen[2,5], Karen Knipping[2,5*].

Table S1 - Observed masses vs theoretical masses as shown in figure 2

Protein		Theoretical mass (Da)	Observed mass (Da)
BLGA*		18363.2	18364.8
BLGB**		18277.1	18277.0
BLGWT	BLGA		18363.6
	BLGB		18278.0

**BLGA*

LIVTQTMKGL DIQKVAGTWY SLAMAASDIS LLDAQSAPLR VYVEELKPTP
EGDLEILLQK WENDECAQKK IIAEKTKIPA VFKIDALNEN KVLVLDTDYK
KYLLFCMENS AEPEQSLVCQ CLVRTPEVDD EALEKFDKAL KALPMHIRLS
FNPTQLEEQC HI
Disulfide bridge formation; 66-160, 106-119

***BLGB*

LIVTQTMKGL DIQKVAGTWY SLAMAASDIS LLDAQSAPLR VYVEELKPTP
EGDLEILLQK WENGECAQKK IIAEKTKIPA VFKIDALNEN KVLVLDTDYK
KYLLFCMENS AEPEQSLACQ CLVRTPEVDD EALEKFDKAL KALPMHIRLS
FNPTQLEEQC HI
Disulfide bridge formation; 66-160, 106-119

Theoretical mass (Da) was calculated using PerkinElmer ChemDraw Professional 17.1 based on the sequences above which were deduced from Uniprot P02754.

Table S2 - Protocol for applying a triple coating on a bare fused silica capillary

#	Pressure (psi)	Duration (min)	Component	Event
1	30	30	NaOH	Activation silanol groups
2	30	20	MQ	Rinse
3	20	20	BGE	Rinse
4	10	30	PB	Application 1st layer
5	30	10	MQ	Rinse
6	10	30	DS	Application 2nd layer
7	30	10	MQ	Rinse
8	10	30	PB	Application 3rd layer
9	30	45	MQ	Rinse
10	20	45	BGE	Rinse

MQ = MilliQ water, BGE = background electrolyte, PB = polybrene, DS = dextran sulphate

Proteins may adsorb to the negatively charged inner wall of BFS capillaries, complicating CE separation and reproducibility. Hence, to avoid adsorption, a 90-110 cm capillary was coated with a triple layer of PB, DS and PB. The separation of BLGA and BLGB was optimized using different BGE compositions (0.25, 0.5, 1 and 2 M acetic and formic acid) and by varying other parameters like capillary temperature, separation voltage and injection volume. A 90 cm triple layer coated capillary, maintained at 25°C, using a BGE of 1 M formic acid, 30 kV separation voltage and an injection volume of 0.86% of the total capillary volume were found to be optimal for the analysis of BLGA and BLGB.

Table S3 - Determination optimal resolution BLGWT separation

BGE	M	Resolution (90cm)	Resolution (110cm)	Commentary (90cm)	Commentary (110cm)
AA	0.25	n.c.	0.39	No separation	
	0.5	n.c.	0.84	No separation	
	1	n.c.	0.82	No separation	
	2	0.59	n.c.		Very low intensity
FA	0.25	0.67	0.63		
	0.5	0.78	n.c.		Retention time > 60 min
	1	0.88	n.c.		Retention time > 60 min
	2	n.c.	n.c.	Very low intensity	Retention time > 60 min

n.c. = not calculated

Resolution was calculated using formula: 2 (t_{r2}-t_{r1})/(W_2+W_1), in which t_r = retention time, W = elution peak width of first eluting peak (1) or second eluting peak (2). Minimal capillary length of ~85 cm is necessary to able to connect the CE to the MS equipment. AA = acetic acid, FA = formic acid.

Table S4 - Observed masses of intact BLG and conjugates

Theoretical masses intact protein			Observed masses intact protein					
			BLGWT		*t=0*		*t=3*	
Lactose units	*BLGA*	*BLGB*	*BLGA*	*BLGB*	*BLGA*	*BLGB*	*BLGA*	*BLGB*
0	18367.3	18281.2	18363.4	18276.2	18362.2	18275.4		
1	18691.6	18605.5			18687.4	18604.5		
2	19015.9	18929.8			19010.4	18925.5		
3	19340.2	19254.1					19335.6	19249.8
4	19664.5	19578.4					19659.7	19573.7
5	19988.8	19902.7					19983.8	19897.7
6	20313.1	20227.0					20308.0	20222.9
7	20637.4	20551.3					20632.0	20547.9
8	20961.7	20875.6					20955.2	20872.3
9	21286.0	21199.9					21279.6	21196.7
10	21610.3	21524.2						
11	21934.6	21848.5						
12	22258.9	22172.8						
13	22583.2	22497.1						
14	22907.5	22821.4						
15	23231.7	23145.6						
16	23556.0	23469.9						
17	23880.3	23794.2						
18	24204.6	24118.5						
19	24528.9	24442.8						
Range lactose units / protein:			0		0-2		3-9	
			t=8		*t=16*		*t=24*	
Lactose units	*BLGA*	*BLGB*	*BLGA*	*BLGB*	*BLGA*	*BLGB*	*BLGA*	*BLGB*
0	18367.3	18281.2						
1	18691.6	18605.5						
2	19015.9	18929.8						
3	19340.2	19254.1						
4	19664.5	19578.4						
5	19988.8	19902.7						
6	20313.1	20227.0						
7	20637.4	20551.3						
8	20961.7	20875.6	20958.7					
9	21286.0	21199.9	21282.0	21194.3				
10	21610.3	21524.2	21605.3	21517.6				
11	21934.6	21848.5	21930.3	21842.5				
12	22258.9	22172.8	22254.4	22167.7	22255.4	22168.7		
13	22583.2	22497.1	22578.6	22492.4	22578.6	22494.2	22576.5	22490.3
14	22907.5	22821.4	22902.7	22817.3	22901.8	22817.4	22901.7	22817.9
15	23231.7	23145.6	23228.3	23142.6	23228.0	23140.4	23227.0	23140.4
16	23556.0	23469.9			23551.0	23464.3	23551.4	23463.5
17	23880.3	23794.2						
18	24204.6	24118.5						
19	24528.9	24442.8						
Range lactose units / protein:			8-15		12-16		13-16	

Table S5 - Percental increase glycated peptides during first 8 hours

Percental increase of identified glycated peptides at t = 3 relative to t = 0;

1	2	3	4	5	6	7	8	9	10	11	12	13	14	15	16	17	18	19	20	21	22	23	24	25	26	27	28	29	30
L	I	V	T	Q	T	M	K	G	L	D	I	Q	K	V	A	G	T	W	Y	S	L	A	M	A	A	S	D	I	S
22.5	22.5	22.5	22.5	22.5	22.5	22.5	22.5																						

31	32	33	34	35	36	37	38	39	40	41	42	43	44	45	46	47	48	49	50	51	52	53	54	55	56	57	58	59	60
L	L	D	A	Q	S	A	P	L	R	V	Y	V	E	E	L	K	P	T	P	E	G	D	L	E	I	L	L	Q	K
										8.5	8.5	8.5	8.5	8.5	8.5	8.5	8.5	8.5	8.5	8.5	8.5	8.5	8.5	8.5	8.5	8.5	8.5	8.5	8.5

61	62	63	64	65	66	67	68	69	70	71	72	73	74	75	76	77	78	79	80	81	82	83	84	85	86	87	88	89	90
W	E	N	D	E	C	A	Q	K	K	I	I	A	E	K	T	K	I	P	A	V	F	K	I	D	A	L	N	E	N
										14.4	14.4	14.4	14.4	14.4	35.9	35.9							17.0	17.0	17.0	17.0	17.0	17.0	17.0

91	92	93	94	95	96	97	98	99	100	101	102	103	104	105	106	107	108	109	110	111	112	113	114	115	116	117	118	119	120
K	V	L	V	L	D	T	D	Y	K	K	Y	L	L	F	C	M	E	N	S	A	E	P	E	Q	S	L	V	C	Q
17.0										2.0	4.4	4.4	4.4	4.4	4.4	4.4	4.4	4.4	4.4	4.4	4.4	4.4	4.4	4.4	4.4	4.4	4.4	4.4	4.4

121	122	123	124	125	126	127	128	129	130	131	132	133	134	135	136	137	138	139	140	141	142	143	144	145	146	147	148	149	150
C	L	V	R	T	P	E	V	D	D	E	A	L	E	K	F	D	K	A	L	K	A	L	P	M	H	I	R	L	S
4.4	4.4	4.4	4.4												62.4	62.4	62.4	118.9	118.9	118.9									

151	152	153	154	155	156	157	158	159	160	161	162
F	N	P	T	Q	L	E	E	Q	C	H	I

Percental increase of identified glycopeptides at t = 8 relative to t = 3;

1	2	3	4	5	6	7	8	9	10	11	12	13	14	15	16	17	18	19	20	21	22	23	24	25	26	27	28	29	30
L	I	V	T	Q	T	M	K	G	L	D	I	Q	K	V	A	G	T	W	Y	S	L	A	M	A	A	S	D	I	S
97.4	97.4	97.4	97.4	97.4	97.4	97.4	97.4																						

31	32	33	34	35	36	37	38	39	40	41	42	43	44	45	46	47	48	49	50	51	52	53	54	55	56	57	58	59	60
L	L	D	A	Q	S	A	P	L	R	V	Y	V	E	E	L	K	P	T	P	E	G	D	L	E	I	L	L	Q	K
										196.6	196.6	196.6	196.6	196.6	196.6	196.6	196.6	196.6	196.6	196.6	196.6	196.6	196.6	196.6	196.6	196.6	196.6	196.6	196.6

61	62	63	64	65	66	67	68	69	70	71	72	73	74	75	76	77	78	79	80	81	82	83	84	85	86	87	88	89	90
W	E	N	D	E	C	A	Q	K	K	I	I	A	E	K	T	K	I	P	A	V	F	K	I	D	A	L	N	E	N
38.9	38.9	38.9	38.9	38.9	38.9	38.9	38.9	38.9																					

91	92	93	94	95	96	97	98	99	100	101	102	103	104	105	106	107	108	109	110	111	112	113	114	115	116	117	118	119	120
K	V	L	V	L	D	T	D	Y	K	K	Y	L	L	F	C	M	E	N	S	A	E	P	E	Q	S	L	V	C	Q
	8.9	8.9	8.9	8.9	8.9	8.9	8.9	8.9	8.9	50.1	29.9	29.9	29.9	29.9	29.9	29.9	29.9	29.9	29.9	29.9	29.9	29.9	29.9	29.9	29.9	29.9	29.9	29.9	29.9

121	122	123	124	125	126	127	128	129	130	131	132	133	134	135	136	137	138	139	140	141	142	143	144	145	146	147	148	149	150
C	L	V	R	T	P	E	V	D	D	E	A	L	E	K	F	D	K	A	L	K	A	L	P	M	H	I	R	L	S
29.9	29.9	29.9	29.9																		27.4	27.4	27.4	27.4	27.4	27.4	27.4		

151	152	153	154	155	156	157	158	159	160	161	162
F	N	P	T	Q	L	E	E	Q	C	H	I

Glycated residues are shown rimmed. Alternatively, the percental increase is represented by the green intensity.

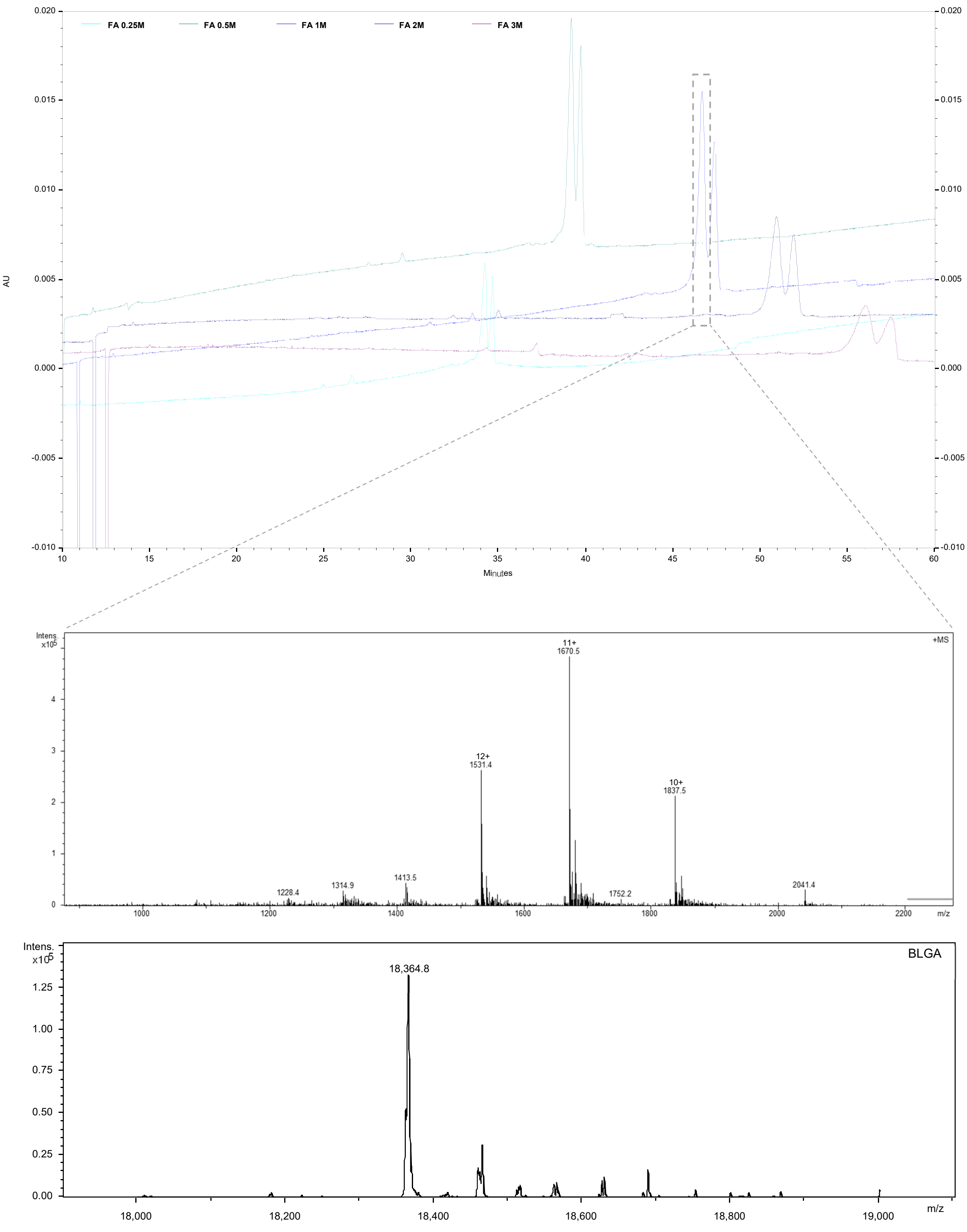

Figure S1 - CE-MS analysis of BLG. (Upper) Electropherogram. (Middle) Profile spectrum. (Bottom) Deconvoluted spectrum of BLGA. Using a triple layer coating and a capillary of 90 cm with a BGE of FA 1M best separation of BLGA and BLGB was observed (BLGA was the first peak to elute, secondly BLGB).
